# Pre-exposure prophylaxis in Guyana: linking key populations and other vulnerable groups

**DOI:** 10.1016/j.ijregi.2025.100823

**Published:** 2025-12-09

**Authors:** Alisa Khan, Tariq Jagnarine

**Affiliations:** 1 Rajiv Gandhi University of Science and Technology, Georgetown, Guyana; 2 National AIDS Programme Secretariat, Ministry of Health, Georgetown, Guyana

**Keywords:** HIV prevention, PrEP, Key populations, Stigma, Guyana, Health care access

## Abstract

**Objectives:**

Despite the introduction of pre-exposure prophylaxis (PrEP) in Guyana as an effective HIV prevention tool, its uptake remains low among key and vulnerable populations, such as men who have sex with men, sex workers, and transgender individuals. This study assessed awareness, uptake, and barriers to PrEP among seronegative individuals in Guyana and proposed strategies to improve accessibility and use.

**Methods:**

A cross-sectional, mixed-method study was conducted among 90 seronegative adults across Regions 3, 4, 6, and 10. Quantitative data were collected via structured questionnaires, whereas qualitative insights were gathered through interviews and focus group discussions. Statistical analyses (chi-square) and thematic coding were applied.

**Results:**

Among participants, 59% were aware of PrEP, primarily through media sources (43%), whereas only 18% had ever been offered PrEP by a health care provider. None of the participants were active users. The key barriers identified were limited access (48%), fear of side effects (27%), and inadequate provider engagement. However, 57% expressed willingness to use PrEP and 74% would recommend it to peers.

**Conclusions:**

PrEP awareness in Guyana is moderate but constrained by systemic barriers, misinformation, and health care inaccessibility. Strengthening provider training, integrating PrEP into routine services, and community outreach are essential to improve uptake.

## Highlights

• Moderate pre-exposure prophylaxis (PrEP) awareness (59%) but zero current usage among participants.
• Limited provider engagement—only 18% were ever offered PrEP services.
• Main barriers: poor access (48%) and fear of side effects (27%).
• Strong preference (79%) for long-acting injectable PrEP options.
• High willingness to recommend PrEP (74%), showing strong advocacy potential.

## Introduction

HIV remains one of the leading public health challenges in Guyana and the wider Caribbean. Although Guyana has achieved notable progress in HIV testing, treatment, and viral suppression, new infections continue to occur, particularly, among key and vulnerable populations, such as men who have sex with men (MSM), transgender persons, and female sex workers (FSWs) [1]. HIV is a retrovirus that progressively weakens the immune system, rendering individuals more susceptible to opportunistic infections and, in its advanced stage, leading to AIDS [2].

Pre-exposure prophylaxis (PrEP) is a biomedical HIV prevention strategy involving the use of antiretroviral drugs by individuals who are HIV-negative to reduce their risk of infection. When taken consistently, PrEP reduces the risk of acquiring HIV through sexual contact by approximately 99% and through injection drug use by about 74% [3]. International guidelines from the World Health Organization (WHO) and the Centers for Disease Control and Prevention (CDC) recommend PrEP as an essential component of combination HIV prevention, alongside behavioral, biomedical, and structural interventions [3,4].

Guyana introduced PrEP in 2019 through the Ministry of Health’s National AIDS Programme Secretariat (NAPS) in collaboration with civil society partners, as part of a broader effort to scale-up HIV prevention [5,6]. Despite this milestone, uptake has been slow. National data indicate that only 86 individuals were accessing PrEP by 2023, highlighting persistent gaps in awareness, availability, and adherence [5]. Several barriers—including limited provider knowledge, stigma, misconceptions about PrEP, and accessibility constraints—continue to undermine use among those most at risk [[6], [7], [8]].

Global evidence supports PrEP as a key intervention for HIV epidemic control. Studies in Kenya, Uganda, and South Africa demonstrate that integrating PrEP into sexual and reproductive health services and family planning clinics enhances accessibility and uptake [9,10]. Similarly, countries such as Australia have observed substantial declines in HIV incidence after PrEP scale-up and integration into routine sexual health care [11,12]. However, successful implementation requires context-specific adaptation and community engagement to address unique sociocultural and health system barriers [13,14].

Guyana’s HIV epidemic remains concentrated, with the 2022 Integrated Behavioural and Biological Survey reporting prevalence rates of 2.2% among FSWs, 1.8% among MSM, and 11.8% among transgender persons—figures considerably higher than the national average [15]. Region 4 (Demerara–Mahaica) continues to account for the majority of cases due to its dense urban population and high concentration of key populations [1,15].

Given the high burden of HIV among these groups and the limited reach of PrEP, there is an urgent need to understand the determinants influencing awareness, acceptability, and uptake of PrEP in Guyana. This study, therefore, seeks to assess levels of PrEP awareness and use, identify barriers to access, and develop practical strategies to strengthen PrEP implementation through multi-sectoral collaboration. The findings will provide valuable evidence to guide national HIV prevention programming and contribute to achieving the Joint United Nations Programme on HIV/AIDS (UNAIDS) 95-95-95 targets and the broader goal of ending AIDS as a public health threat by 2030 [14].

## Research questions

1. What is the current level of awareness and uptake of PrEP among individuals who are HIV-negative and key populations in Guyana?
2. What are the major barriers influencing PrEP access, adherence, and acceptability?
3. What innovative strategies can be used to increase PrEP uptake across key and vulnerable populations?
4. What role can health care providers, community leaders, and civil society organizations play in promoting PrEP use?
5. What mechanisms can be developed to ensure sustainability and long-term adherence to PrEP within Guyana’s public health system?

## Research objectives

### General objective

To assess the awareness, uptake, barriers, and attitudes toward PrEP among seronegative populations in Guyana and propose strategies for improving PrEP access and use.

### Specific objectives

1. To assess the current level of PrEP awareness and uptake quantitatively using a structured questionnaire among key and general populations.
2. To identify key barriers that challenge PrEP uptake qualitatively through in-depth interviews and focus group discussions (FGDs).
3. To develop innovative and evidence-based strategies to improve linkage to PrEP services and address identified barriers.
4. To engage relevant stakeholders—including health professionals, community organizations, and policymakers—in implementing these strategies.
5. To explore approaches to ensure sustainability and long-term adherence to PrEP through continuous monitoring, education, and support mechanisms.

## Literature review

### Global overview of HIV and PrEP

Worldwide, HIV remains a major public health concern, with an estimated 39 million people living with HIV as of 2023 [14]. Despite progress in treatment and prevention, new infections persist, especially among populations with limited access to prevention services. The introduction of PrEP has revolutionized HIV prevention by providing a biomedical intervention that complements behavioral and structural approaches [4].

The WHO first recommended oral PrEP containing tenofovir disoproxil fumarate in 2015 for individuals at substantial risk of infection [4]. Subsequent guidance introduced event-driven PrEP for MSM and the dapivirine vaginal ring as a female-controlled option [16]. The CDC confirmed that consistent PrEP use reduces sexual transmission by up to 99% and transmission through injection drug use by 74% [3].

Evidence from multiple studies supports its effectiveness and safety. Fonner et al. demonstrated that PrEP significantly reduces HIV acquisition across populations without increasing adverse events [17]. Similarly, Smith et al. [12] found that increases in PrEP coverage in the United States were associated with corresponding declines in new HIV diagnoses, underscoring PrEP’s importance in epidemic control.

### Regional and Caribbean context

In the Caribbean, HIV continues to disproportionately affect key populations—including MSM, transgender individuals, and sex workers—driven by stigma and social marginalization [13]. Although treatment access has improved, PrEP implementation remains uneven across the region. Studies in Jamaica and Trinidad and Tobago report low awareness, cost constraints, and reluctance among health care providers to prescribe PrEP [13].

Guyana, an early PrEP adopter in 2019, continues to experience low uptake. By 2023, only 86 individuals were accessing PrEP despite expanded testing and treatment coverage [5]. Local research found that 60% of key population respondents had never heard of PrEP and often confused it with post-exposure prophylaxis, with stigma and perceived side effects as major deterrents [7]. Although Guyana has initiated community-based programs and provider training, efforts remain urban-centered, leaving hinterland and rural populations underserved [6]. PrEP scale-up, therefore, requires access expansion and targeted education to address misconceptions and social determinants of health-seeking behavior.

### Awareness and acceptability of PrEP

Awareness and acceptability are essential for uptake. Global PrEP awareness varies widely—from over 80% in some US populations to less than 30% in sub-Saharan Africa [10]. A multi-country study in Kenya, Uganda, and South Africa revealed that although young people viewed PrEP positively, fear of stigma and inadequate counseling limited consistent use [9].

In Guyana, awareness is moderate but understanding remains limited. The 2022 Integrated Behavioural and Biological Survey reported HIV prevalence of 2.2% among FSWs and 11.8% among transgender persons, yet few could accurately define or access PrEP [15]. This highlights the need for awareness campaigns grounded in behavioral change communication rather than mere information dissemination.

### Barriers to uptake

Barriers to PrEP implementation include health care provider knowledge gaps, side-effect concerns, limited access, and stigma. Adherence remains a key determinant of effectiveness; Landovitz et al. [18] demonstrated that long-acting injectable cabotegravir reduced HIV incidence by 66% compared with daily oral regimens.

In Guyana, service concentration in Region 4 limits availability in remote regions such as 1, 7, 8, and 9 [5]. Facility-based stigma and misconceptions—such as beliefs that PrEP encourages promiscuity—further discourage uptake [8]. Addressing these barriers requires health system reform and sociocultural sensitivity in service delivery.

### Innovative strategies and implementation models

Worldwide, countries have adopted innovative strategies to expand PrEP access. Integration into primary care and reproductive health services, task-shifting to nurses and pharmacists, and mobile health (mHealth) tools have proven effective [19]. Kenya’s youth-friendly clinics and digital adherence systems enhanced access and retention [10]. The US “Ready, Set, PrEP” program further demonstrated how pharmacy-based and mail-order models increase national uptake [20].

Guyana has begun adopting similar community-oriented strategies. The National AIDS Programme Secretariat’s 2023 “PrEP for Sex” campaign provided educational materials, condoms, and self-testing kits to promote awareness [5]. However, sustainable success requires institutional integration, continuous provider training, and structured monitoring systems to track adherence and outcomes.

### Research gap

Although global data affirm PrEP’s effectiveness, its potential in Guyana remains under-realized. Current research highlights limited awareness, access inequities, and systemic barriers, but few studies use mixed methods to examine both quantitative and qualitative determinants of PrEP use. This study addresses that gap by combining survey data with stakeholder insights to inform policy, strengthen multi-sectoral collaboration, and support national progress toward achieving the UNAIDS 95-95-95 goals and eliminating HIV as a public health threat by 2030 [14].

## Methodology

### Research design

A cross-sectional mixed-methods design was used to examine awareness, uptake, and barriers to PrEP among key populations in Guyana. Quantitative data were collected through structured questionnaires and qualitative insights through in-depth interviews and FGDs. This triangulated approach improved reliability and contextual understanding [21].

### Study area and population

The study covered four administrative regions—3 (Esequibo Islands–West Demerara), 4 (Demerara–Mahaica), 6 (East Berbice–Corentyne), and 10 (Upper Demerara–Berbice)—selected for high HIV prevalence and diversity of key populations [15]. Participants were adults who were HIV-negative aged ≥18 years, including MSM, FSWs, sero-discordant couples, and other at-risk individuals.

### Sampling and sample size

A stratified random sampling method ensured proportional representation by risk group and region. A total of 90 participants were enrolled, a feasible exploratory sample allowing variation across strata [22].

### Data collection instruments

Quantitative: A structured questionnaire, adapted from the CDC and WHO PrEP frameworks [3,4], captured demographics, HIV testing history, PrEP knowledge, and behavioral practices.

Qualitative: Nine FGDs (≈eight persons each) and key informant interviews explored perceptions, barriers, and programmatic needs. Sessions lasted ≈45 minutes and were audio-recorded with consent.

### Ethical considerations

Approval was obtained from the institutional review board of Rajiv Gandhi University of Science and Technology and the National AIDS Programme Secretariat. Written informed consent was secured from all participants. Confidentiality was maintained using coded identifiers and private venues, following the WHO (2016) ethical guidance for key population research and the Declaration of Helsinki (2013) [4].

### Data analysis

Quantitative: Data were analyzed in SPSS v26. Descriptive statistics summarized demographics and awareness. Associations between categorical variables were tested using chi-square (χ²) at *P* <0.05.

Qualitative: Transcripts were thematically analyzed using Braun and Clarke’s six-phase framework to identify themes on access, stigma, and system challenges. Triangulation enhanced validity [21].

### Reliability and validity

The questionnaire was pre-tested with 10 respondents outside study sites. Internal consistency achieved a Cronbach’s α ≥0.70, indicating acceptable reliability [23]. For qualitative data, peer debriefing and member checking verified credibility.

### Limitations

Findings are limited by the modest sample size (n = 90) and restriction to four regions, which may affect generalizability. Self-reported behaviors could introduce social desirability bias, although confidentiality and method triangulation mitigated this.

## Results

**Figure 1:**
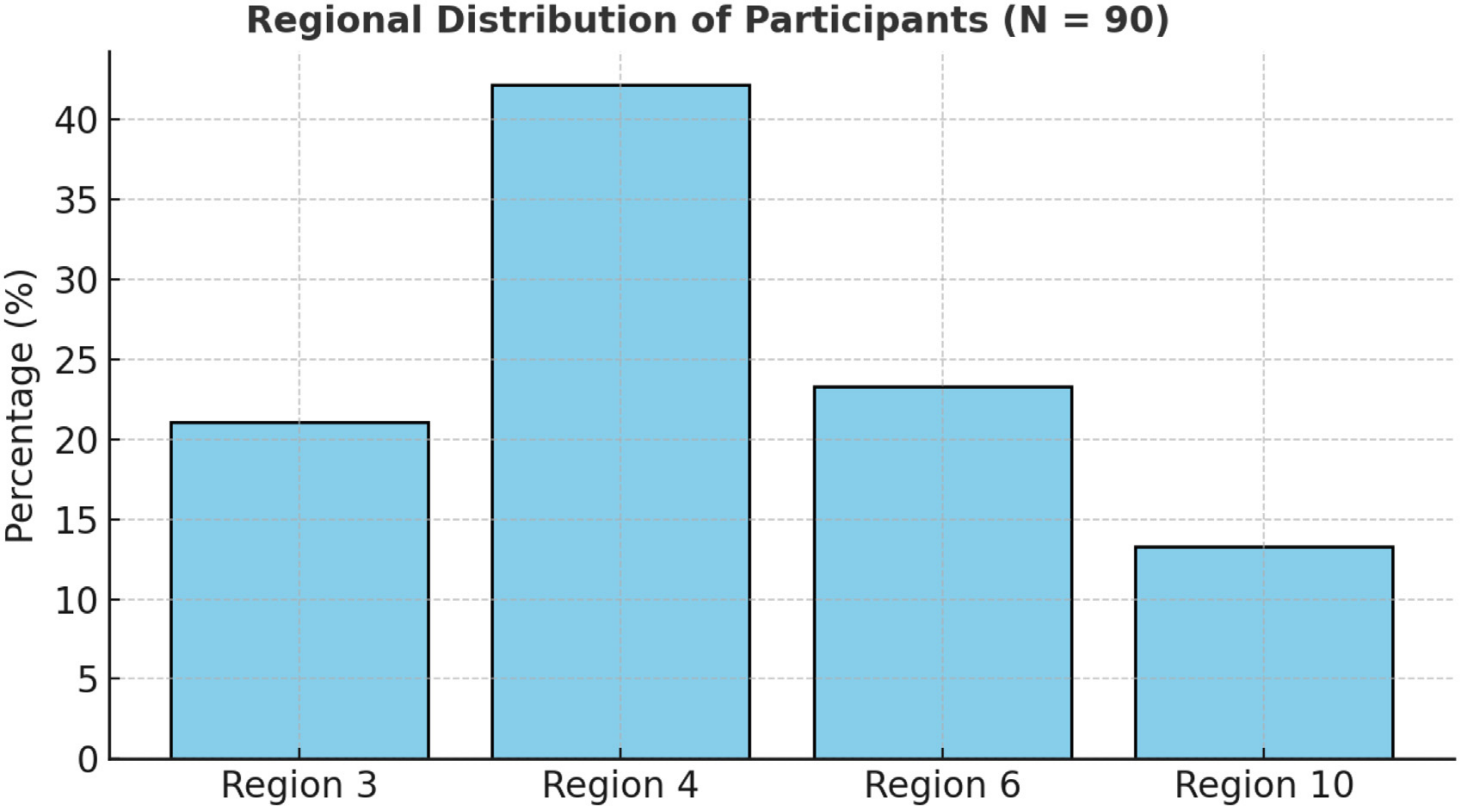
Regional distribution of participants (N = 90). A bar chart showing highest concentration in Region 4 (42.2%), followed by Regions 6, 3, and 10.

**Figure 2:**
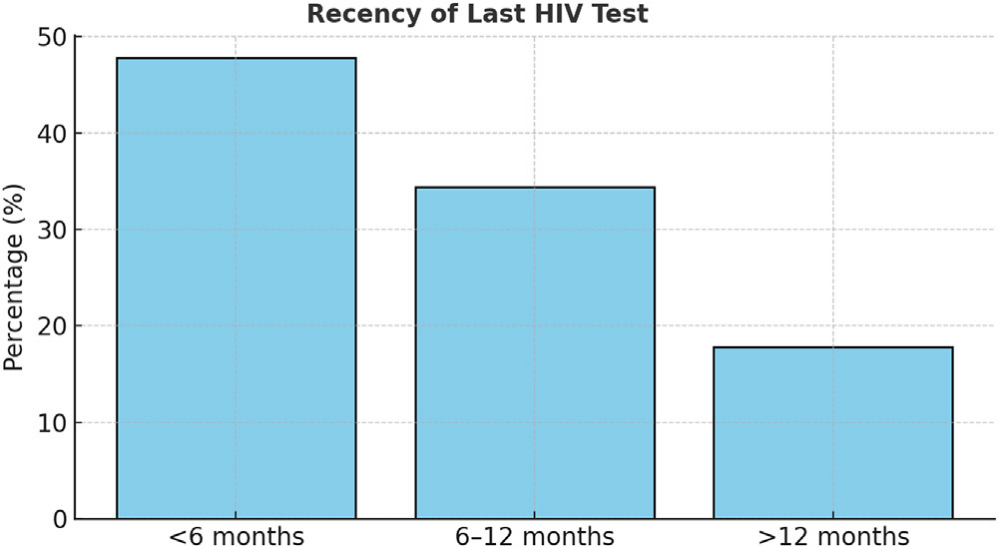
Recency of last HIV test among respondents. A clustered bar chart showing testing within <6 months (47.8%) as most common.

The study included 90 participants who were HIV-negative across four regions of Guyana. The largest proportion were from Region 4 (42.2%), followed by Region 6 (23.3%), Region 3 (21.1%), and Region 10 (13.3%), reflecting the national HIV burden’s geographic distribution [15] (See Figure 1). All participants reported previous HIV testing, with 47.8% tested within the last 6 months, indicating commendable testing frequency and engagement with health care services (See Figure 2). However, condom use remained inconsistent—only 26.7% reported always using condoms—highlighting continuing exposure risks and the importance of supplementary prevention methods, such as PrEP [3,5] (Tables 1 and 2).

**Table 1:** Socio-demographic and regional distribution of participants (N = 90).

Variable	Category	Frequency (n)	Percentage (%)
Region	Region 3 – Essequibo Islands–West Demerara	19	21.1
	Region 4 – Demerara–Mahaica	38	42.2
	Region 6 – East Berbice–Corentyne	21	23.3
	Region 10 – Upper Demerara–Berbice	12	13.3
Gender	Male	48	53.3
	Female	39	43.3
	Transgender	3	3.4
Age group (years)	18-24	25	27.8
	25-34	33	36.7
	35-44	19	21.1
	≥45	13	14.4

Source: PrEP awareness and uptake survey. Guyana, 2025.

**Table 2:** HIV testing history and sexual behavior.

Indicator	Category	Frequency (n)	Percentage (%)
Ever tested for HIV	Yes	90	100.0
Last HIV test	<6 months ago	43	47.8
	6-12 months ago	31	34.4
	>12 months ago	16	17.8
Condom use frequency	Always	24	26.7
	Sometimes	53	58.9
	Never	13	14.4

Source: PrEP awareness and uptake survey. Guyana, 2025.

**Figure 3:**
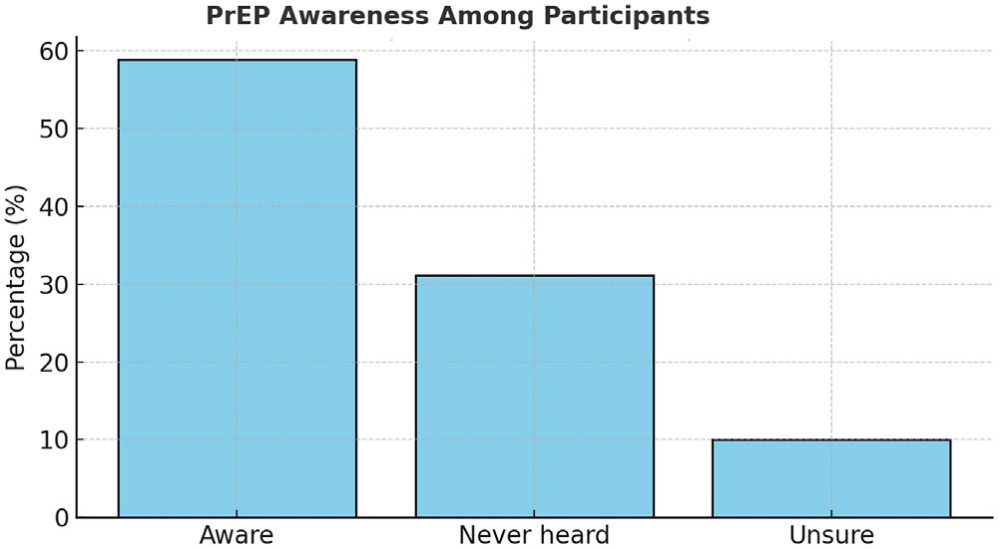
PrEP awareness and understanding. PrEP, pre-exposure prophylaxis. A stacked column chart illustrating 58.9% awareness, with 79.2% accurate understanding.

**Figure 4:**
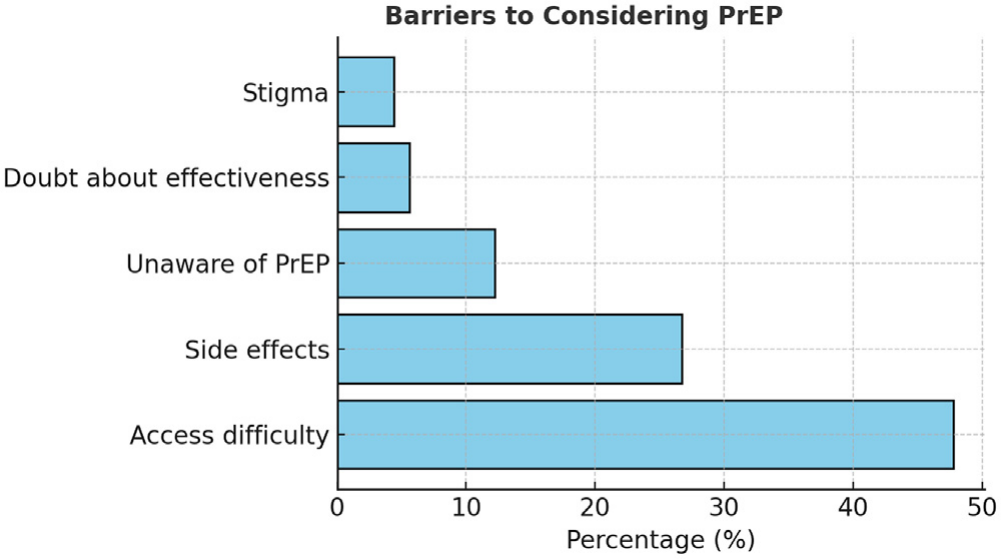
Primary source of PrEP information. PrEP, pre-exposure prophylaxis. A pie chart showing 43.3% from media, 10% from health care providers, 31.1% never heard.

**Figure 5:**
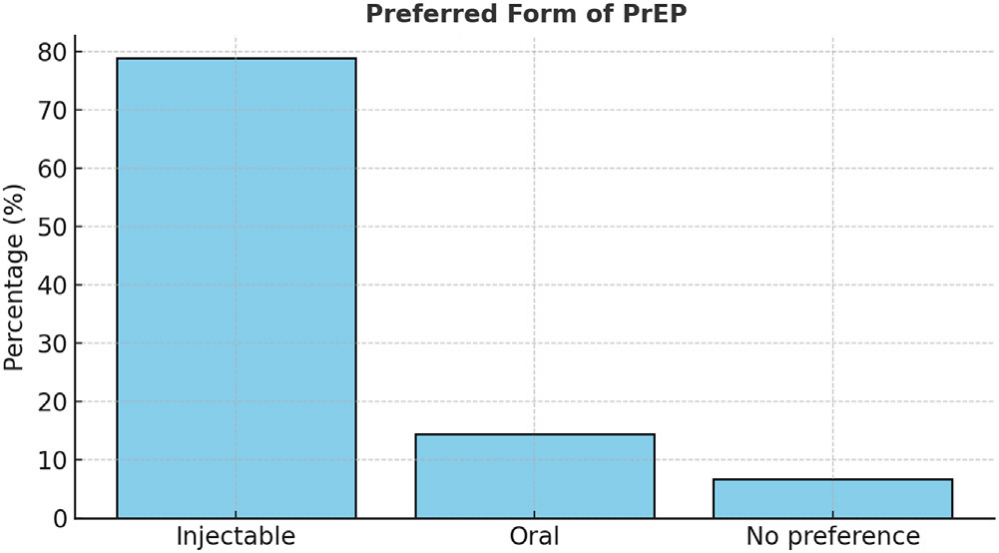
Barriers to considering PrEP. PrEP, pre-exposure prophylaxis. A horizontal bar chart depicting leading reasons: access (47.8%), side effects (26.7%), awareness (12.2%).

PrEP awareness among participants was moderate, with 58.9% indicating familiarity with the intervention, although 31.1% had never heard of it (See Figure 3). Of those aware, 79.2% correctly understood its preventive role, primarily learning through mass media (43.3%), whereas only 10% received information from health care providers (see Figure 4). This demonstrates a limited provider contribution to community PrEP knowledge despite existing guidelines and national promotion strategies [5,6]. Moreover, only 17.8% of respondents had ever been offered PrEP by a health care worker, and none were current users (See Figure 5). These figures expose a substantial gap between national policy and service delivery, reflecting similar provider-related bottlenecks seen in African PrEP implementation studies [9,10] (Tables 3 and 4).

**Table 3:** PrEP awareness, understanding, and sources of information.

Indicator	Category	Frequency (n)	Percentage (%)
Heard of PrEP	Yes	53	58.9
	No	28	31.1
	Unsure	9	10.0
Correct understanding among those aware (n = 53)	Accurate	42	79.2
	Misconception	11	20.8
Main source of PrEP information	Media	39	43.3
	Health care provider	9	10.0
	Community organization	5	5.6
	Friends/Family	6	6.7
	Never heard	28	31.1

PrEP, pre-exposure prophylaxis.

Source: PrEP awareness and uptake survey. Guyana, 2025.

**Table 4:** PrEP access, willingness, and barriers.

Indicator	Category	Frequency (n)	Percentage (%)
Ever offered PrEP by provider	Yes	16	17.8
	No	74	82.2
Current PrEP use	Yes	0	0.0
	No	90	100.0
Willing to consider PrEP	Yes	51	56.7
	No	21	23.3
	Unsure	18	20.0
Main reasons for not considering PrEP	Access difficulty	43	47.8
	Fear of side effects	24	26.7
	Unaware of PrEP	11	12.2
	Doubt about effectiveness	5	5.6
	Stigma	4	4.4

PrEP, pre-exposure prophylaxis.

Source: PrEP awareness and uptake survey. Guyana, 2025.

**Figure 6:**
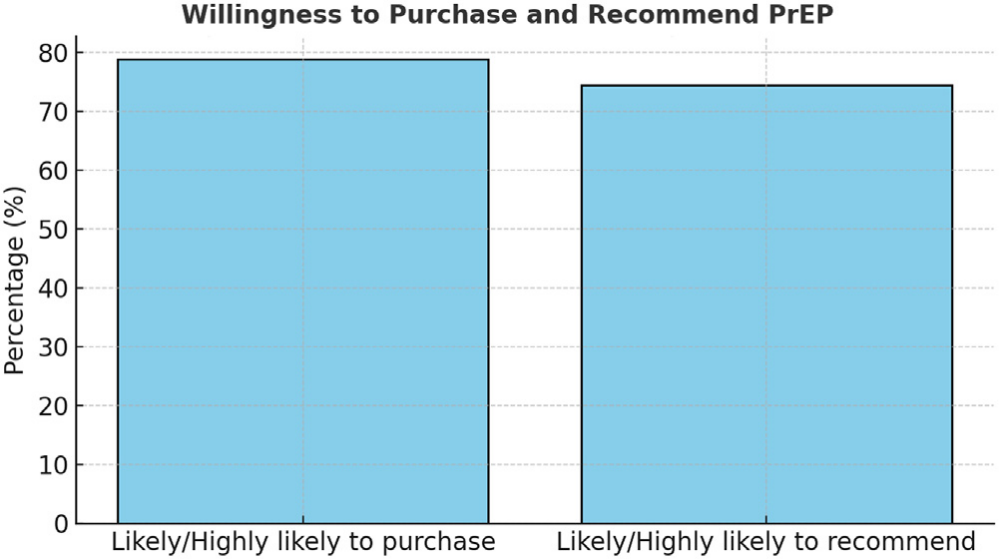
Preferred form of PrEP. PrEP, pre-exposure prophylaxis. A bar chart showing preference for injectables (78.9%) over oral PrEP (14.4%).

When participants were asked about willingness to use PrEP, 56.7% stated they would consider it, 23.3% were unwilling, and 20% were unsure. The main reasons for non-use included difficulty accessing services (47.8%), concerns about side effects (26.7%), limited awareness (12.2%), and skepticism about effectiveness (5.6%). Stigma was a minor but notable factor (4.4%), indicating some social discomfort despite wider acceptance of HIV prevention messaging [7,8]. Awareness of different PrEP formulations was low (37.8%), yet 78.9% of respondents expressed a strong preference for injectable PrEP over oral pills, consistent with global findings on user adherence and convenience [18] (See Figures 5, 6 and Table 5).

**Table 5:** Preferences and attitudes toward PrEP.

Indicator	Category	Frequency (n)	Percentage (%)
Aware of multiple PrEP forms	Yes	34	37.8
	No	56	62.2
Preferred PrEP form	Injectable	71	78.9
	Oral	13	14.4
	No preference	6	6.7
Likely to purchase PrEP	Likely/Highly likely	71	78.9
	Not likely	19	21.1
Likely to recommend PrEP	Likely/Highly likely	67	74.4
	Not likely	23	25.6

PrEP, pre-exposure prophylaxis.

Source: PrEP awareness and uptake survey. Guyana, 2025.

The analysis further revealed that 78.9% of participants were likely or highly likely to purchase PrEP if available and affordable, and 74.4% would recommend it to peers. These results suggest substantial latent demand, which could be harnessed through improved accessibility and counseling. Statistical testing confirmed that the proportion of individuals offered PrEP by providers (17.8%) was significantly below the 50% benchmark (*P* <0.001), underscoring a major systemic shortfall in service promotion. In addition, willingness to recommend PrEP and preference for injectables were significantly above 50% (*P* <0.001), revealing strong advocacy potential and acceptance of long-acting options.

**Figure 7:**
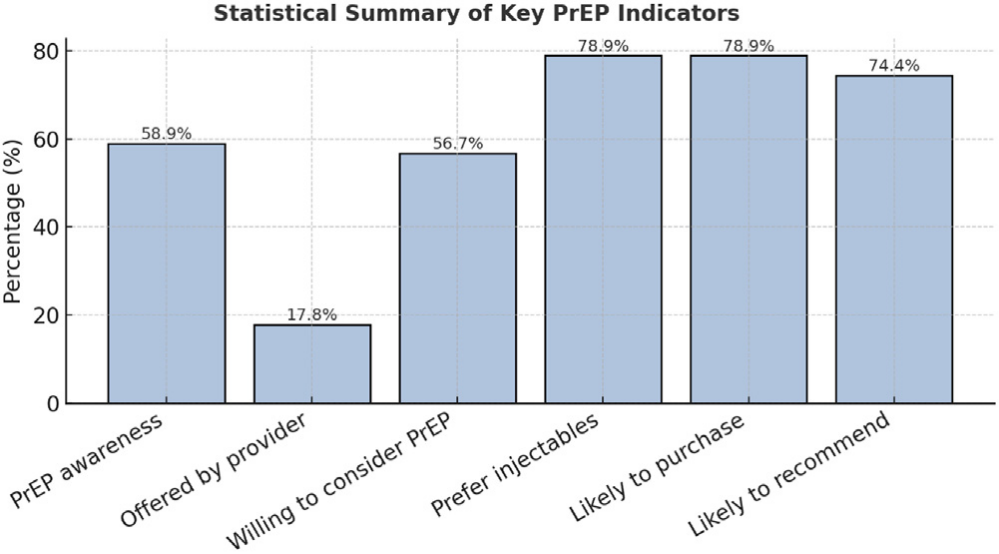
Willingness to purchase and recommend PrEP. PrEP, pre-exposure prophylaxis. A side-by-side bar chart showing 78.9% likely to purchase and 74.4% likely to recommend.

Collectively, these findings indicate a paradox: although PrEP awareness and interest exist, structural barriers—especially limited provider engagement and geographic inequities—impede uptake. The high levels of willingness to use and recommend PrEP demonstrate a readiness among key populations for expansion, provided logistical, informational, and attitudinal barriers are addressed. This mirrors patterns in other middle-income settings where community-led outreach, decentralization, and targeted digital campaigns effectively bridge awareness–uptake gaps [13,19,20] (See Figure 7).

These insights confirm the necessity for Guyana’s National AIDS Programme Secretariat to strengthen provider training; decentralize PrEP services beyond Region 4; and use innovative, youth-friendly, and gender-sensitive communication models. Addressing these gaps will be critical to transforming PrEP awareness into measurable coverage, advancing Guyana’s progress toward the UNAIDS 95-95-95 targets and the 2030 HIV elimination goal [14].

## Discussion

This study provides new insight into the awareness, accessibility, and barriers surrounding PrEP among individuals who are HIV-negative and key populations in Guyana. Although awareness levels were moderate (58.9%), uptake was non-existent, reflecting a persistent disconnect between knowledge and behavior. This finding mirrors trends across low- and middle-income settings, where awareness does not necessarily translate into adoption due to system-level and sociocultural barriers [9,10]. The limited number of participants ever offered PrEP by health care providers (17.8%) suggests a critical service delivery gap within the national response. Similar provider-related constraints have been reported in Kenya, where uncertainty about eligibility and inadequate training hindered PrEP integration into clinical workflows [10].

The strong preference for long-acting injectable PrEP (78.9%) aligns with global findings demonstrating improved adherence and acceptability for cabotegravir compared with daily oral regimens [18]. Participants’ interest in injectable options reflects a preference for discreet, convenient, and low-burden prevention methods—key factors in settings where stigma and privacy concerns remain influential. However, awareness of PrEP’s different formulations was limited (37.8%), suggesting that public education campaigns should emphasize product diversity and individualized options [5].

Accessibility emerged as the dominant barrier, cited by nearly half of respondents (47.8%). This echoes patterns in sub-Saharan Africa, where PrEP awareness is often undermined by logistical challenges, such as supply chain limitations, clinic distance, and urban bias in service distribution [9,13]. In Guyana, the concentration of PrEP services in Region 4 excludes hinterland and riverine populations, perpetuating inequities in HIV prevention [5]. Decentralizing access through regional hospitals, health centers, and community pharmacies could substantially increase uptake, especially among underserved groups.

Although only 4.4% of respondents cited stigma as a direct barrier, qualitative accounts suggested subtle forms of discrimination within health settings—particularly, toward MSM and transgender persons. These findings align with Society Against Sexual Orientation Discrimination’s earlier report [7], which noted that stigma operates more implicitly through health care interactions than through overt rejection. Such hidden stigma may discourage individuals from seeking prevention services or disclosing risk behaviors. Therefore, integrating stigma-reduction training for health care providers remains essential to achieving equitable PrEP access.

Encouragingly, the high willingness to recommend and purchase PrEP (74.4% and 78.9%, respectively) demonstrates community readiness and advocacy potential. These results align with international studies showing that peer-led models and community engagement are effective in scaling PrEP awareness and uptake [10,19]. Harnessing this enthusiasm through structured peer educator programs could amplify outreach, particularly, among youth and key populations.

Overall, these findings underscore the urgent need for a multifaceted strategy combining education, provider training, and service decentralization. The observed gaps indicate that PrEP scale-up in Guyana is not limited by lack of interest but by structural inefficiencies and insufficient integration into routine health care. Expanding access through task-shifting to nurses and pharmacists, digital adherence tools, and public–private partnerships—as demonstrated in other successful models [19,20]—could accelerate national targets.

In alignment with the UNAIDS 95-95-95 agenda and Guyana’s goal of ending AIDS as a public health threat by 2030 [14], PrEP should be positioned as a cornerstone intervention alongside HIV testing, treatment, and condom distribution. Effective PrEP implementation requires a supportive ecosystem—one that links biomedical innovation with community empowerment, sustained financing, and human rights–based health delivery.

“The findings also have significant implications for Guyana’s long-term HIV elimination strategy. Countries that achieved substantial reductions in HIV incidence, such as Australia and parts of the United States, attribute progress to comprehensive PrEP integration, strong provider engagement, and diversified modalities including long-acting injectables [11,12]. Incorporating such models into Guyana’s health system—particularly through decentralized delivery, task-shifting, and integration within sexual and reproductive health services—can accelerate PrEP uptake and complement existing combination prevention efforts. Additionally, embedding PrEP monitoring into national electronic health systems aligns with WHO guidance on standardised surveillance and can support sustained adherence and impact measurement [4,24].”

### Limitations

Although comprehensive, the study’s findings are constrained by the relatively small sample size and limited geographic coverage (four regions). Self-reported data on sexual behavior and PrEP awareness may be affected by recall or social desirability bias. Nonetheless, triangulation of quantitative and qualitative data and ethical safeguards enhanced the credibility of the results [27].

## Recommendations

### Health system strengthening

• Integrate into routine care: Embed PrEP counseling and initiation within HIV testing, antenatal, and family planning services to ensure all at-risk clients are offered PrEP.
• Decentralize access: Expand PrEP provision to regional hospitals, health centers, and community pharmacies—especially in hinterland areas—to bridge service gaps.
• Train healthcare providers: Implement ongoing education on PrEP eligibility, prescription, and follow-up to strengthen provider confidence and uptake [3].

### Community engagement and awareness

• Peer-led outreach: Empower MSM, FSWs, and transgender networks to lead relatable, stigma-free PrEP campaigns.
• Digital and mass media campaigns: Use social media, radio, and community platforms to promote culturally sensitive and youth-focused messaging.
• Gender-sensitive communication: Tailor content to women, adolescents, and minority groups to enhance inclusivity and trust.

### Programmatic and policy reform

• Introduce injectable PrEP (long-acting cabotegravir [CAB-LA]): Expedite regulatory approval and logistics for CAB-LA in line with user preference.
• Strengthen monitoring and evaluation: Use electronic systems to track PrEP initiation, adherence, and retention.
• Remove financial barriers: Ensure PrEP is free or subsidized through public–private and non-governmental organization partnerships.

### Research and continuous improvement

• Longitudinal evaluation: Monitor long-term adherence, behavioral outcomes, and program effectiveness to inform national policy.
• Targeted research: Investigate gender- and age-specific barriers among youth, women, and sero-discordant couples to refine outreach strategies.

“The introduction of long-acting PrEP should be prioritised, given strong user preference and global evidence that injectable cabotegravir significantly reduces HIV incidence compared to oral regimens [18]. Preparing for CAB-LA rollout will require updating national guidelines, strengthening cold-chain logistics, and implementing targeted training for clinicians and nurses. Countries in sub-Saharan Africa have demonstrated that such scale-up is feasible when paired with community mobilization, pharmacy-based access, and digital adherence interventions [10,13,25]. Guyana can adapt these models to ensure equitable access across urban and hinterland regions.”

### Future steps and application

The findings of this study highlight several priority areas for strengthening PrEP implementation in Guyana. Future efforts should focus on integrating PrEP counseling and initiation into existing service points—such as HIV testing, antenatal, and family planning clinics—to capitalize on routine client contact and reduce missed prevention opportunities [5,26]. Expanding PrEP delivery to regional hospitals, primary health care centers, and community pharmacies is essential to address the urban–rural gap and improve access for hinterland and indigenous populations [1,5]. Given the strong preference for long-acting formulations, national preparation for the rollout of injectable cabotegravir (CAB-LA) should be accelerated through provider training, supply chain strengthening, and updated clinical guidelines [4,18]. Digital adherence tools, mobile health interventions, and peer-led community mobilization—shown to enhance uptake and reduce stigma worldwide—represent practical strategies for local adaptation [10,25]. Continued research is also required to monitor real-world adherence; evaluate gender- and age-specific barriers; and assess PrEP outcomes among adolescents, young adults, and women in sero-discordant relationships [7,9]. These future steps will support the Ministry of Health and partners in scaling a comprehensive, equitable, and sustainable PrEP program aligned with the UNAIDS 95-95-95 targets and the 2030 goal of ending AIDS as a public health threat in Guyana [14], [28].

## Conclusion

This study assessed awareness, attitudes, and barriers influencing the uptake of PrEP among key and vulnerable populations in Guyana. Although global evidence supports PrEP as a highly effective HIV prevention tool, its local implementation remains limited. Although over half of participants were aware of PrEP, none reported current use—revealing a clear gap between knowledge and practice.

Limited access, low provider engagement, and concerns about side effects were the primary barriers, whereas stigma appeared less severe than previously reported. The strong preference for long-acting injectable PrEP highlights readiness for discreet and convenient prevention options. The high willingness to recommend and purchase PrEP further suggests latent demand that can be leveraged through education, decentralization, and community-led delivery models.

The findings underscore the need for integration of PrEP into HIV testing and family planning services, expansion to regional facilities, and enhanced provider training. Collaborative efforts among the Ministry of Health, NAPS, UNAIDS, and civil society will be vital to advancing the UNAIDS 95-95-95 targets and achieving the goal of ending HIV transmission in Guyana by 2030.

Overall, this study provides baseline evidence to guide PrEP policy and programming. Strengthening public trust, empowering communities, and sustaining investment will be critical to positioning PrEP as a cornerstone of Guyana’s HIV prevention response.

## Funding

This study received no external funding and was conducted as part of academic research under the Rajiv Gandhi University of Science and Technology in collaboration with the Ministry of Health, Guyana.

## Ethical approval statement

Ethical approval was granted by the institutional review board of the Rajiv Gandhi University of Science and Technology, with authorization from the NAPS, Ministry of Health. Written informed consent was obtained from all participants, and confidentiality was maintained in accordance with the Declaration of Helsinki (2013) and WHO [4] ethical guidelines for key population research.

## Author contribution

The research was analyzed and discussed by Dr Tariq JAgnarine, and data collection by Dr Khan.

## Declaration of competing interest

The authors have no competing interests to declare.
